# A Rotational Slurry Bioreactor Accelerates Biodegradation of A-Fuel in Oil-Contaminated Soil Even under Low Temperature Conditions

**DOI:** 10.3390/microorganisms8020291

**Published:** 2020-02-20

**Authors:** Yuna Miyoshi, Jo Okada, Tomotaka Urata, Masaki Shintani, Kazuhide Kimbara

**Affiliations:** 1 Department of Engineering, Graduate School of Integrated Science and Technology, Shizuoka University, 3-5-1 Johoku, Naka-ku, Hamamatsu, Shizuoka 432-8561, Japan; miyoshi.yuna.14@shizuoka.ac.jp (Y.M.); okada.joh.15@shizuoka.ac.jp (J.O.); urata.tomotaka.16@shizuoka.ac.jp (T.U.); 2 Research Institute of Green Science and Technology, Shizuoka University, 836 Ohya, Suruga-ku, Shizuoka, Shizuoka 422-8529, Japan

**Keywords:** A-fuel oil, *Rhodococcus*, bioaugmentation, rotational slurry bioreactor

## Abstract

An effective bioaugmentation system for oil-contaminated soil under low-temperature conditions was developed with a rotational slurry bioreactor. Mixtures of two *Rhodococcus* oil-degraders, strain A and C, which are officially permitted to be used in bioaugmentation in Japan, were inoculated and A-fuel oil was added to a final concentration of 2500 and 5000 mg/kg-slurry. Decomposition tests were carried out for the inoculated samples and non-inoculated samples by rotating at 15 °C, the annual average temperature of Japan. The residue of A-fuel oil and the number of bacteria were measured every two days. After 6 days of treatment, more than 95% of the oil was removed in the inoculated samples, which was more than three times faster than a previous degradation experiment without rotation. A semi-continuous treatment was performed by removing 90% of the treated slurry, then adding the same amount of contaminated slurry into the system without additional degraders. Ninety-four percent of A-fuel oil was successfully degraded after 6 days by this repeated treatment. This could drastically reduce the cost of preparing the degraders. Strikingly, semi-continuous treatment showed oil removal in the non-inoculated samples, indicating that the rotational slurry conditions could efficiently promote biodegradation by indigenous degraders. Our rotational slurry bioreactor accelerated the removal of oil contamination without adding further degraders provides an efficient and cost-effective method of removal of A-fuel oil using a semi-continuous system, which can be used in practical applications in areas with a cooler climate.

## 1. Introduction

Soil contamination caused by oil spills is a global problem [1,2]. Bioaugmentation with oil-degrading bacteria has been used as an effective treatment to clean up the contaminated soil [3,4,5]. However, the certainty and efficiency of bioaugmentation is not necessarily stable, because various environmental conditions, including soil composition, water content, temperature, and indigenous microbes, influence the feasibility of this treatment [6,7,8]. Among these conditions, temperature was one of the most important factors, because the microbial process could be further slowed down in cooler or cold environments [7,8]. Therefore, the isolation and usage of microbial degraders adapted to cold environments have been performed in several reports [9,10,11,12,13,14,15]. In our previous study, two *Rhodococcus* strains, A and C, could degrade the A-fuel oil (500 mg/kg-soil) at lower temperatures (15 and 10 °C) as well as at 30 °C [16]. These two *Rhodococcus* strains have been permitted for bioaugmentation by the Environment Agency in Japan based on the guidelines for environmental risk assessment on bioremediation (bioaugmentation) to prefectural governments. Thus, these strains could be used for bioaugmentation in cool areas including northern parts of the Japanese main island (where the annual mean temperatures were around 10–15 °C, according to the tables of climatological normal at Japan Meteorological Agency, https://www.data.jma.go.jp/obd/stats/data/en/normal/normal.html).

The water content of soil was also an important factor for bioaugmentation (articles were listed in the reviews [6,17]), and indeed, our previous study showed that slurry treatment of the model contaminated soil was efficient for bioaugmentation [18]. This treatment might increase contact frequency between bacterial cells (degrader) and their substrate (contaminants) due to the presence of water.

Agitation is an important step for the effective removal of contaminants in bioaugmentation, although it requires large equipment [19]. Several kinds of rotational bioreactors have been developed to accelerate composting organic wastes by mixing the indigenous degraders and their substrates [20,21,22]. These reactors were usually used at 25–30 °C and low temperature always negatively affects biodegradation rates [23]. In the present study, to assess whether the slurry treatments and rotation could effectively work on bioaugmentation at low temperatures, we developed a lab-scale rotational slurry bioreactor imitating a concrete mixing system that can be scaled up and used in the field to remediate contaminated soils. The above oil-degraders were used, which can degrade the oil even at low temperatures, and the removal efficiencies of A-fuel oil were compared using the lab-scale rotational slurry bioreactor in different conditions.

## 2. Materials and Methods

### 2.1. Bacterial Strains and Culture Conditions

*Rhodococcus erythropolis* strain A and *R. erythropolis* strain C [16] were permitted as microbial degraders for use in bioaugmentation by the Japanese Ministry of Environment in 2011 (Guidelines for Environmental Risk Assessment on Bioremediation). These strains were grown overnight at 30 °C in 3-fold diluted Lysogeny Broth (1/3 LB) (tryptone 3.3 g/L, yeast extract 1.7 g/L and NaCl 5.0 g/L) [24]. Phosphate-buffered minimal salt medium [W medium: KH_2_PO_4_ (1.7 g/L), Na_2_HPO_4_ (9.8 g/L), (NH_4_)_2_SO_4_ (1.0 g/L), MgSO_4_∙7H_2_O (0.1 g/L), FeSO_4_∙7H_2_O (0.95 mg/L), MgO (10.75 mg/L), CaCO_3_ (2.0 mg/L), ZnSO_4_∙7H_2_O (1.44 mg/L), CuSO_4_∙5H_2_O (0.25 mg/L), CoSO_4_∙7H_2_O (0.28 mg/L), H_3_BO_3_ (0.06 mg/L), and concentrated HCl (51.3 mL/L)] were used for the minimum medium [24]. The A-fuel oil, a Japanese heavy oil with the lowest viscosity, was added as a sole carbon source.

### 2.2. Preparation of Model Slurry

Model slurry samples were prepared using a modified version of the method of our previous report [16]. In brief, two different commercial horticultural soils were used: Akadama soil (Fujimikogyo Co. Ltd., Shizuoka, Japan), which is a volcanic ash soil in the Kanto area of Japan (non-heat-treated), and Sibame sand (Fujimikogyo) which is a dried sand (heat-treated). Akadama soil (180 g) and Shibame sand (120 g) were mixed. The percentages of sand, silt, and clay of the model soil were 85.3%, 1.40%, and 13.3%, respectively. The texture of the model soil is loamy sand, according to the Soil Survey Manual of the United States Department of Agriculture [25]. The total carbon and total nitrogen of the soil samples were 3.0 g/kg and 0.30 g/kg, respectively. The weight of the dry soil was measured, and 200 mL distilled W medium was added to a final water content of >100%. A-fuel oil was added to the slurry with 2500 mg/kg-slurry or 5000 mg/kg-slurry. The composition of n-alkanes in the A-fuel oil is shown in Table 1. The slurry and A-fuel oil were not sterilized.

### 2.3. A Rotational Slurry Bioreactor

**Figure 1:**
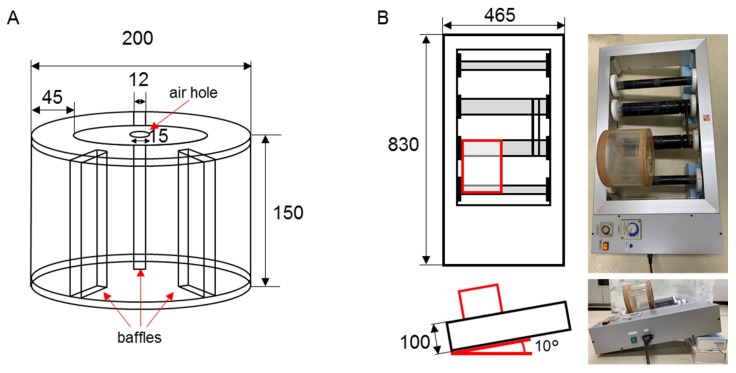
A 5 L rotational slurry bioreactor panel (**A**) and rotator and reactor with model slurry panel (**B**). The digits indicate size of reactor (mm). The system was put in a 15 °C incubator with 10° tilt. The red box indicates the reactor.

A 5 L rotational bioreactor was made of acrylic cylinder (5-mm thickness) with three baffles (Figure 1A). The lid has a 15-mm air hole (Figure 1A). A Plate Mix Mill PMM-20 (NSkouken, Co. Ltd., Aichi, Japan) was used for rotation with 12 rpm and a 10° angle (Figure 1B). The rotator and reactor with model slurry were mixed in the above conditions in a 15 °C incubator after the addition of A-fuel into the model slurry in the bioreactor for 24 h (premixing, before starting the test of biodegradation). The 10^6^–10^9^ (colony forming units (CFUs)/g-slurry) of A-fuel oil degraders were inoculated into the slurry sample, mixed for 30 min, and then the sample was collected. The 10^8^–10^9^ CFUs/g-slurry of degraders were cultured in 0.8 L of 1/3 LB.

### 2.4. Biodegradative Assays in the Rotational Slurry Bioreactor

Three different soil slurry samples were prepared: (i) rotated at 12 rpm (the standard rate of agitation in concrete mixing system) and degraders were not inoculated, (ii) rotated at 12 rpm and the strains A and C were inoculated, (iii) non-rotated and the strains were inoculated. Each sample was incubated at 15 °C. The extraction of the remaining A-fuel oil from the samples was performed at 0, 2, 4, and 6 days after starting the test. As for the first three sampling points (0, 2, and 4 days), 1-g slurry samples were collected, whereas 3 g of slurry samples were collected at 6 days. Afterwards, 5 mL (for the samples collected at 0 and 2 days) or 3 mL (for the samples collected at 4 and 6 days) hexane was added to each sample in capped 15 mL glass test tubes, respectively. The A-fuel oil was extracted by shaking at 200 rpm for 60 min. After being left standing for 30 min, 1 mL of the supernatant was subjected to a step to remove water and debris by Na_2_SO_4_ addition and centrifugation. Then, the resultant sample was subjected to a gas chromatograph system equipped with a FID detector (GC-2014, Shimadzu Corp., Kyoto, Japan) and an autosampler (AOC-20i, SHIMADZU). The GC column was DB-1 (Agilent Technologies Japan, Tokyo, Japan) and helium was used as a carrier gas at a flow rate of 3.58 mL/min, with a temperature increase of 20 °C/min from 40 (initial temperature) to 250 °C and 10 °C/min from 250 to 320 °C (final temperature), with holds of 5 min at the initial and final temperatures. CDS-Lite ver.5 (LAsoft Ltd., Chiba, Japan) was used to analyze the chromatography data. The standard curve was made with 250, 500, and 1000 mg/L of A-fuel oil in hexane.

The numbers of total bacteria and degraders of A-fuel oil in the model soil slurry were counted as follows: one gram of soil slurry sample collected at each sampling day was resuspended with 3 mL of sterilized phosphate buffer saline (PBS) [26] with 3 μL of Tween-80 and was mixed by vortex. The supernatant of the sample was serially diluted by PBS and spread on 1/3 LB plates and W plates with A-fuel oil (supplemented as a vapor by putting 10 μL of the oil on the lid of plate). After incubating the plates at 30 °C for a few days, the colonies on the plate were counted.

### 2.5. Semi-Batch Degradative Assays in the Rotational Slurry Bioreactor

**Figure 2:**
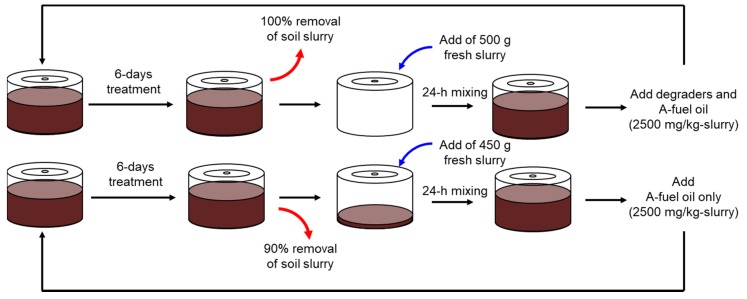
One cycle of semi-batch degradative assays in the rotational slurry bioreactor. Control assays (upper) with 100% removal of the soil slurry after 6-day treatment and semi-batch assays (lower). Three cycles were done.

As for the semi-batch degradative assays, 10% of the model slurry (50 g) was left after 6-day treatments of biodegradation with A-fuel oil degraders, and 90% of fresh slurry (450 g) was added. After 24 h of mixing, A-fuel oil was added into the slurry with its final concentration being 2500 mg/kg-slurry (no degraders were inoculated). As a control, 100% of the slurry was removed and then 100% fresh slurry was added. After 24 h of mixing, the fresh A-fuel oil (2500 mg/kg-slurry) and its degraders (10^8^ cells/g-slurry) were inoculated (Figure 2). The remaining A-fuel oil was measured as described above.

## 3. Results

### 3.1. Biodegradation of A-Fuel Oil was Much Faster in a Rotational Slurry Bioreactor

**Figure 3:**
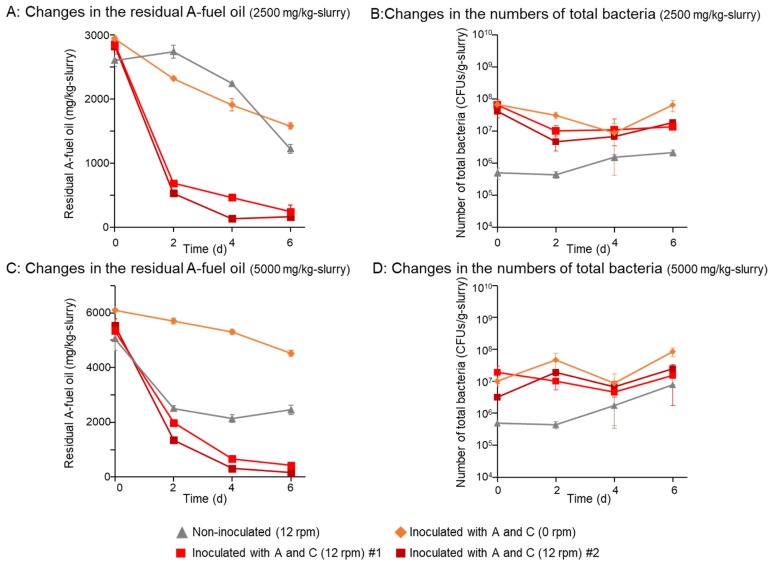
Biodegradation of 2500 panels (**A**,**B**) or 5000 mg/kg-slurry panels (**C**,**D**) of A-fuel oil in rotational slurry bioreactors. Panels (**A**,**C**) show changes in the residual A-fuel oil in 6 days. Panels (**B**,**D**) show changes in the numbers of total bacteria and A-fuel oil degraders (CFU/g-slurry) in 6 days. Two independent assays were performed for the samples inoculated with degraders (strains A and C) at 12 rpm rotation. Standard deviations of triplicate data are shown.

Biodegradation assays of A-fuel oil (2500 mg/kg-slurry) were performed in rotational slurry bioreactors. Using 12-rpm rotation and inoculation with *Rhodococcus erythropolis* strains A and C, 98% of the oil was removed in 6 days (Figure 3A). In contrast, more than 48% of the oil remained in the non-inoculated samples with rotation or the inoculated samples without rotation (Figure 3A). In the samples inoculated with degraders, 10^7^–10^8^ CFUs/g slurry of total bacteria were detected and the numbers of them did not change during the assays (Figure 3B). The level for the sample without inoculation was around 10^5^–10^6^ CFUs/g-slurry (Figure 3B).

Similarly, 90% of A-fuel oil were removed in rotational slurry bioreactors in 6 days, even when the concentration of A-fuel oil increased to 5000 mg/kg-slurry, whereas more than half of them remained in the rotated sample without inoculation with degraders or the inoculated sample without rotation (Figure 3C). The numbers of total bacteria in the samples inoculated with degraders were around 10^7^–10^8^ CFUs/g-slurry, whereas those in the sample without inoculation increased from 10^5^ to 10^7^ cells/g slurry in 6 days (Figure 3D). We performed these experiments several times, and similarly, the A-fuel oil removal was always faster with the inoculation with degraders and rotation of the bioreactor (Figures S1 and S2). However, the number of total bacteria was different at the end of the various experiments (Figures S1 and S2).

### 3.2. Semi-Batch Degradative Assays in the Rotational Slurry Bioreactor

**Figure 4:**
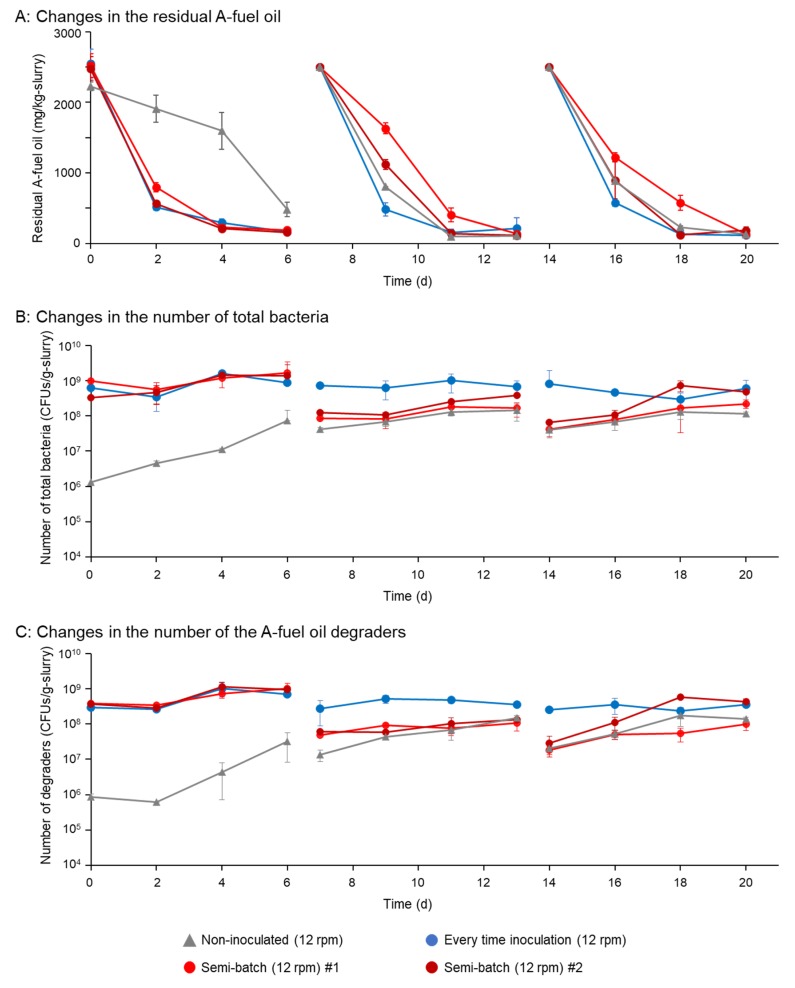
Semi-batch degradative assays of 2500 mg/kg-slurry of A-fuel oil in rotational slurry bioreactors inoculated with 10^8^ CFUs/g-slurry of degraders. Panel (**A**) shows changes in the residual A-fuel oil in three cycles (each cycle was 6 days of treatment). There were 24 h of mixing time between two cycles. Panels (**B**,**C**) show changes in the numbers of total bacteria (B) and A-fuel degraders (C), respectively. Two independent assays were performed for the samples with one-time inoculation of the degraders. Standard deviations of triplicate data are shown.

Semi-batch degradative assays of A-fuel oil (2500 mg/kg-slurry) were performed in rotational slurry bioreactors. For the first cycle, for the samples inoculated with degraders, more than 95% of the oil was removed. In the second and third cycles, the removal rate was faster in the sample with inoculation with degraders than those of semi-batch samples (Figure 4A). In the six-day treatment in each cycle, however, more than 95% of the oil was removed in both samples (Figure 4A). Interestingly, 80% of the oil was removed in the sample without inoculation with degraders at first cycle (Figure 4A). In the second and third cycles, more than 95% of the oil was removed in those samples without inoculation (Figure 4A). As for the changes in the numbers of total bacteria or oil degraders, 10^9^ CFUs/g-slurry of total bacteria and 10^8^ CFUs/g-slurry of oil degraders were found in the samples inoculated with degraders in the first cycle. In the inoculated samples, these numbers were similarly found in the second and third cycles (Figure 4B,C). In the semi-batch samples, the numbers of the total bacteria and oil degraders were at around 10^8^ CFUs/g-slurry and 10^7^ CFUs/g-slurry at the beginning of the cycle (Figure 4B,C). They increased to 10^8^–10^9^ CFUs/g-slurry during 6 days of treatment (Figure 4B,C). In the samples without inoculation with degraders, the numbers of total bacteria and oil degraders increased from 10^6^ to 10^8^ CFUs/g-slurry (Figure 4B,C).

**Figure 5:**
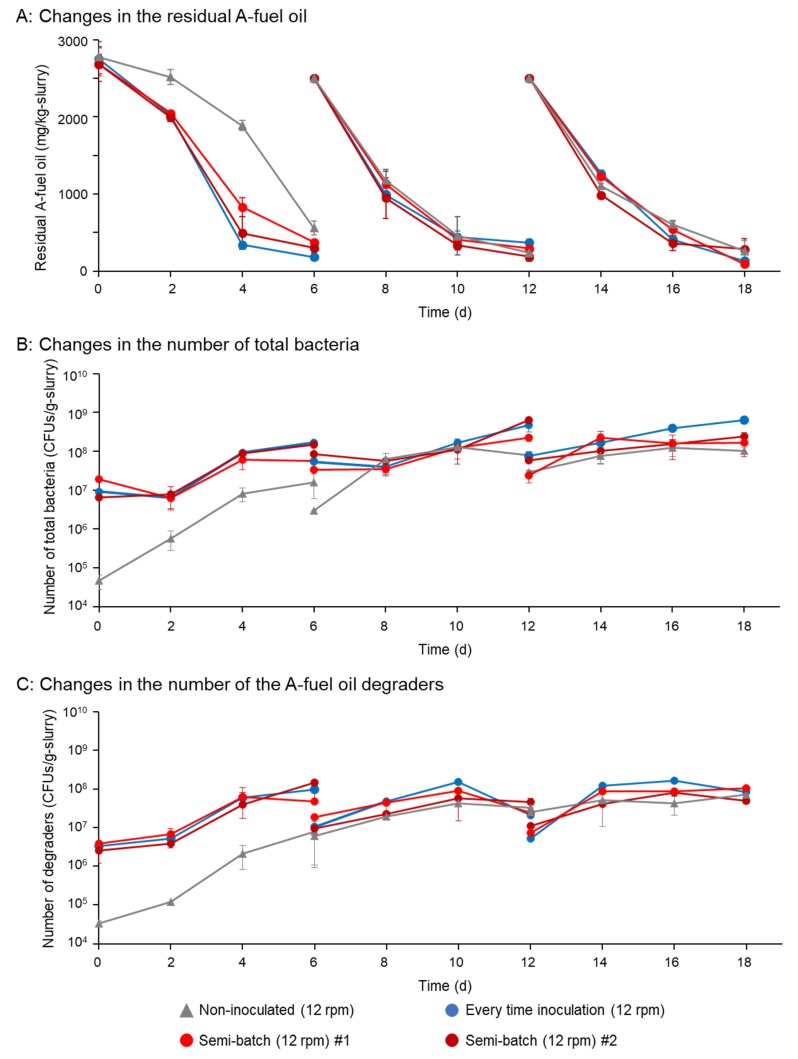
Semi-batch degradative assays of 2500 mg/kg-slurry of A-fuel oil in rotational slurry bioreactors inoculated with 10^6^ CFUs/g-slurry of degraders. The interval 24-h mixing process was omitted. Panel (**A**) shows changes in the residual A-fuel oil in three cycles (each cycle was 6 days of treatment). There were 24 h of mixing time between two cycles. Panels (**B**,**C**) show changes in the numbers of total bacteria (B) and A-fuel degraders (C), respectively. Two independent assays were performed for the samples with one-time inoculation of the degraders. Standard deviations of triplicate data are shown.

To improve the degradative treatment of A-fuel oil in the above semi-batch, we reduced the initial amount of inoculation from 10^8^ to 10^6^ CFUs/g-slurry and omitted the 24 h interval for the mixture of the remaining and fresh slurry. The reduction in the number of inoculated bacteria and omitting the re-inoculation reduced the amount of media for cultivation from 2.4 L to 8 mL. The removal rate of the A-fuel oil during the first cycle was lower than that of the above semi-batch degradative treatment with the inoculation of 10^8^ CFUs/g-slurry (Figure 4A and Figure 5A). In the second and third cycle, the removal rates of the oil were at a similar level to the above treatment with the inoculation of 10^8^ CFUs/g-slurry (Figure 4A and Figure 5A). The numbers of total bacteria and oil degraders increased from 10^6^ to 10^7^ CFUs/g-slurry in the first cycle, and then 10^6^ to 10^8^–10^9^ CFUs/g-slurry in the second and third cycles, which represented similar levels to those in the above treatments (Figure 5B,C). Notably, the numbers of total bacteria and oil degraders in the sample without inoculation increased from 10^4^ to 10^7^ CFUs/g-slurry in the first cycle, and then 10^6^ to 10^7^–10^8^ CFUs/g-slurry in the second and third cycles (Figure 5B,C).

## 4. Discussion

*Rhodococcus erythropolis* strains A and C could degrade A-fuel oil under low-temperature conditions [16]. They effectively remove the oil (80% of the 500 mg/kg-slurry of the oil) from the soil in 12 days of treatment at 15 °C [16]. In the present study, more than 95% of the oil could be removed during a half period of the treatment time (in 6 days), under the rotational slurry conditions tested in this study, and regardless of the type and number of bacteria present in the samples. There are a large number of reports that have investigated removal of oil from soil samples by bioaugmentation [27,28,29,30,31,32]. In their reports, however, it usually took more than 40 days for degradation, although their experimental conditions, including concentrations of oil (2500–50,000 mg/kg soil), types of soils and scale of reactions (0.1–2.5 kg of soil), were different [27,28,29,30,31,32]. In comparison with these reports, our system with rotation and slurry treatment could drastically accelerate the biodegradation of oil contamination in the soil sample. There have been reports that slurry treatments are effective for bioaugmentation [6,17] and have been previously reviewed [23,33]. A rotating biological contractor (RBC) is also known as an efficient system for wastewater treatment [34]. In most cases, the rotation and slurry treatments were effective in the removal of contamination, probably due to increase in the contact between degraders and their substrate, and supplying oxygen in the slurry. However, treatments with these rotating bioreactors at a low temperature have not been reported, which is important when using the system outside in cooler climate areas.

Semi-batch degradative assays successfully showed that one-time inoculation was enough for the removal of A-fuel oil from the soil (Figure 4). Strikingly, the initial inoculation amount of the degraders could be reduced to at least 1/100 and the interval mixing process could be omitted (Figure 4 and Figure 5). This fact indicates that the time and energy costs for the cultivation of degraders could be saved, which is probably important to scale up our system. Furthermore, the reduction in inoculates in the soil could make the impact of inoculating on the soil smaller, while these degraders are permitted as microbial degraders for use in bioaugmentation in open systems.

Interestingly, the removal of A-fuel was observed in batch and semi-batch biodegradative assays, even though no degraders were inoculated (Figure 3, Figure 4 and Figure 5). In the batch analyses, fluctuations in the amount of the remaining oil were found in the non-inoculated samples (Figure 3, Figures S1 and S2). These phenomena were due to the volatility of A-fuel oil, which could be enhanced by rotation. The number of indigenous degraders for A-fuel oil slightly increased in our system because the slurry and A-fuel oil were not sterilized. The numbers of total bacteria fluctuated in different experimental lots (Figures S1 and S2), probably due to using non-sterilized slurry and oil. In the semi-batch assays, the indigenous degraders greatly increased their abundance and the efficient removal of A-fuel oil was also found in the non-inoculated samples (Figure 4 and Figure 5). Therefore, in any case, the indigenous degraders could increase their abundance by rotation, although we have not identified these degraders so far. This fact indicates that slurry treatment and rotation could increase the abundance and activity of indigenous degrader(s), which were also beneficial points of our system.

The systems proposed in the present study with two degraders, officially allowed to be used in bioaugmentation by the Japanese Ministry of the Environment, could be used in applications with concrete mixing transport trucks in cooler climate areas. In the present study, the in-depth mechanism was still unclear as to why and how the slurry treatment and rotation could accelerate the removal of the A-fuel oil. The aerobic degradation of alkanes is usually initiated by the alkane hydroxylase, which converts alkanes to alkanols by consuming oxygen [35]. Recently, one of alkane degraders, relatively close to strains A or C, *R. jostti* strain RHA1, was shown to possess a three-component alkane hydroxylase consisting of alkane 1-monooxygenase, rubredoxin, and rubredoxin reductase [36]. Although the molecular mechanism was still unclear regarding how the strains A and C could degrade A-fuel oil, the two degraders notably had the homologous gene sets in their genome sequences. Therefore, the degradative pathways of these two degraders were probably similar to the previously known one, and oxygen could be one of important factors for the biodegradation of A-fuel oil. Further study of our two strains, including transcriptional levels of *alkB* genes at different temperatures, will reveal the molecular mechanisms in how they could degrade the A-fuel oil in the slurry at lower temperatures. These aspects will be important for more effective bioaugmentation in cooler climates.

## Figures and Tables

**Table 1:** Composition of n-alkanes in 2500-mg A-fuel oil.

	Numbers of Carbon																
	10	11	12	13	14	15	16	17	18	19	20	21	22	23	24	25	26
**Concentration in 2500-mg A-fuel oil (mg)**	27	67	76	134	231	330	341	293	258	209	172	131	90	63	38	25	17

## Supplementary Materials

The following are available online at https://www.mdpi.com/2076-2607/8/2/291/s1. Figure S1. Biodegradation of 2500 mg/kg-slurry of A-fuel oil in rotational slurry bioreactors (results of another lots, #3,4,5,6). Panels A and C showed changes in the residual A-fuel oil in 6 days. Panels B and D showed changes in the numbers of total bacteria and A-fuel oil degraders (CFU/g-slurry) in 6 days. Two independent assays were performed for the samples inoculated with degraders (strains A and C) at 12 rpm rotation. Standard deviations of triplicate data are shown. Figure S2. Biodegradation of 5000 mg/kg-slurry of A-fuel oil in rotational slurry bioreactors (results of another lots, #3,4,5,6). Panels A and C showed changes in the residual A-fuel oil in 6 days. Panels B and D showed changes in the numbers of total bacteria and A-fuel oil degraders (CFU/g-slurry) in 6 days. Two independent assays were performed for the samples inoculated with degraders (strains A and C) at 12 rpm rotation. Standard deviations of triplicate data are shown.
