# Supplementary material for: A Rotational Slurry Bioreactor Accelerates Biodegradation of A-Fuel in Oil-Contaminated Soil Even under Low Temperature Conditions
Source: Microorganisms. 2020 Feb 20;8(2):291. doi: 10.3390/microorganisms8020291 (PMC7074909; doi:10.3390/microorganisms8020291)
Supplement: Supplementary file 1 [file microorganisms-08-00291-s001.pdf]

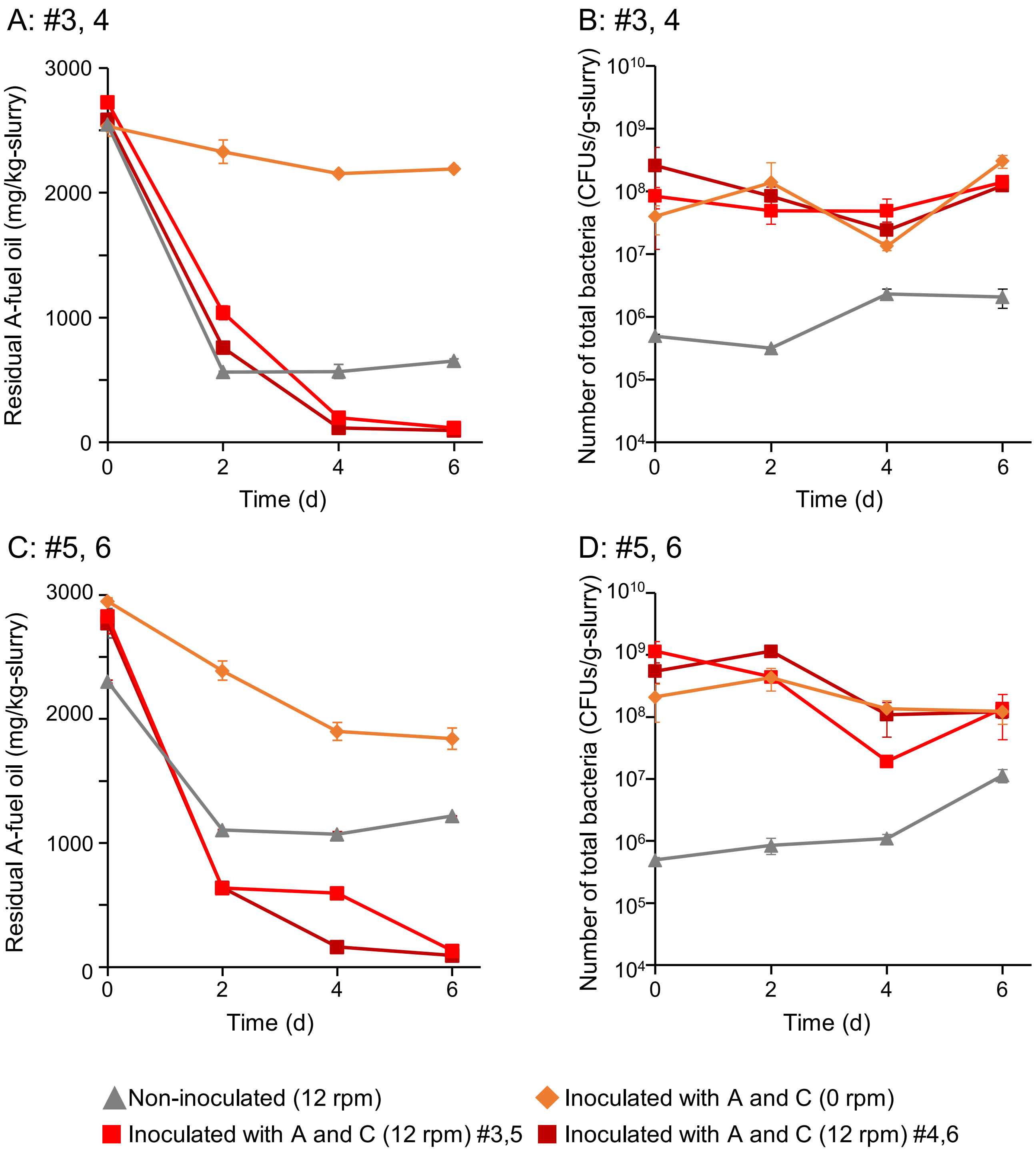

Figure S1. Biodegradation of 2500 mg/kg-slurry of A-fuel oil in rotational slurry bioreactors (results of another lots, #3,4,5,6). Panels A and C showed changes in the residual A-fuel oil in 6 days. Panels B and D showed changes in the numbers of total bacteria and A-fuel oil degraders (CFU/g-slurry) in 6 days. Two independent assays were performed for the samples inoculated with degraders (strains A and C) at 12 rpm rotation. Standard deviations of triplicate data are shown.

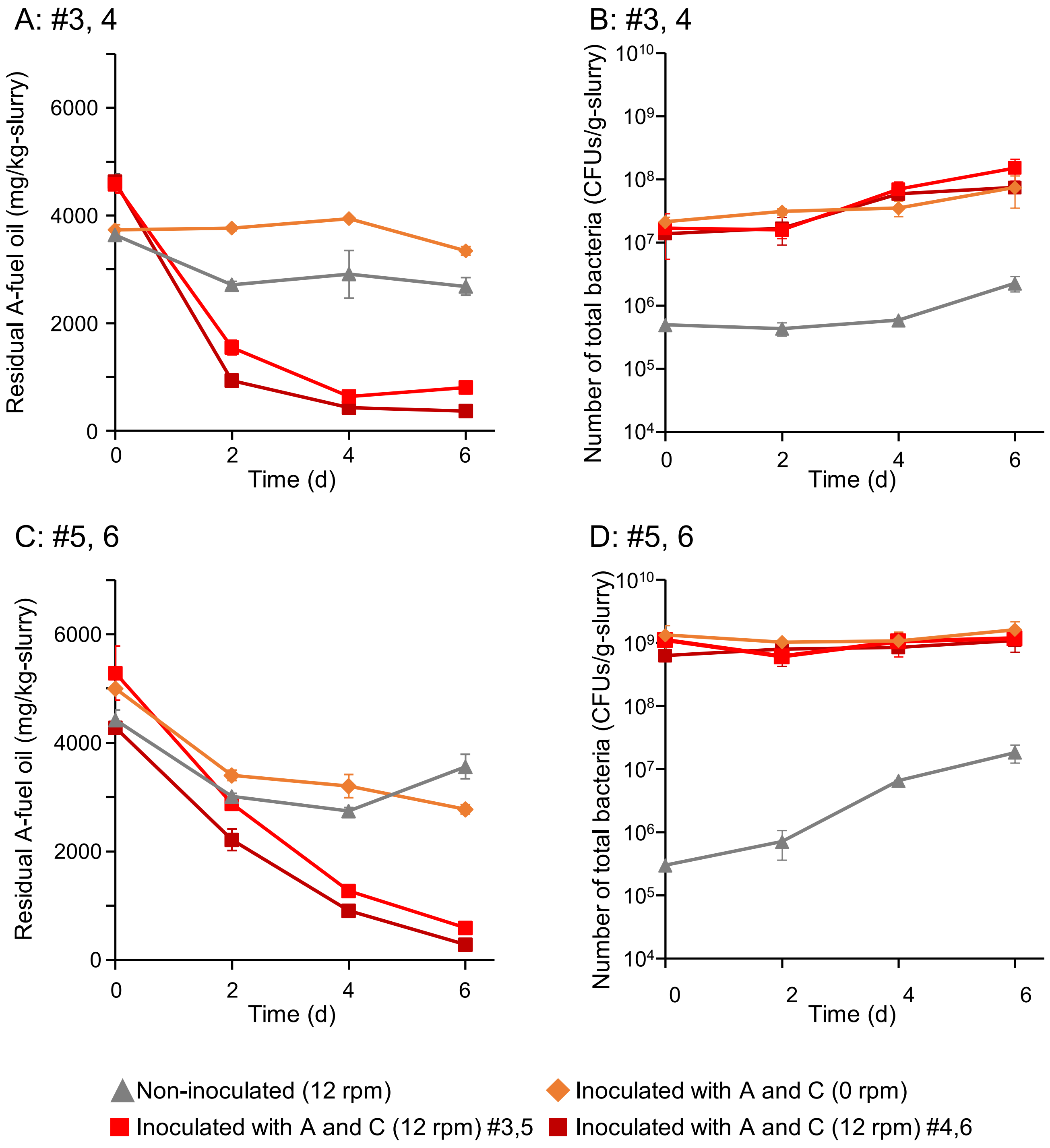

Figure S2. Biodegradation of 5000 mg/kg-slurry of A-fuel oil in rotational slurry bioreactors (results of another lots, #3,4,5,6). Panels A and C showed changes in the residual A-fuel oil in 6 days. Panels B and D showed changes in the numbers of total bacteria and A-fuel oil degraders (CFU/g-slurry) in 6 days. Two independent assays were performed for the samples inoculated with degraders (strains A and C) at 12 rpm rotation. Standard deviations of triplicate data are shown.
